# Hahnemannian Association of Pennsylvania; Report of Proceedings

**Published:** 1887-12

**Authors:** Wm. Jefferson Guernsey

**Affiliations:** Secretary


### HAHNEMANNIAN ASSOCIATION OF PENNSYLVANIA.

### Report of the Proceedings.

The second stated meeting was held in the Continental Hotel Tuesday evening, November 8th, 1887, Dr. Mahlon Preston, of Norristown, presiding. Present, all the active members, one honorary member, and eight visitors.

Dr. C. Carleton Smith read a paper on *Spongia tosta.*

Discussion:

*Dr. Smith:* "Oranges should never be allowed to children suffering from croup. In my experience they usually aggravate the disease, so much so that I have made it an invariable rule to forbid them. When this aggravation is marked it is a characteristic of *Spong.*

*Dr. Mahlon Preston:* "Your paper referred to the *Spongia* amelioration from food or drink; *Anacardium* has an amelioration of stomach symptoms by eating."

*Dr. Robert Farley:* "I have verified that symptom personally."

*Dr. William Jefferson Guernsey:* "*Iod.* and *Puls.* also have that indication, and *Cupr.* has relief of cough from drinking."

*Dr. John V. Allen:* "*Anac.* has nausea and vomiting from cold drinks."

*Dr. Preston:* "At one time I was subject to attacks of pain in the right side of the abdomen which would last for days. It was always better by eating, and on this I took *Anac.* It helped me greatly for several attacks, but finally ceased to do any good, when I discovered that it was the flatulence which was relieved by eating (being constantly annoyed by passing it except after meals). On this indication I took *Teucrium* (which has that symptom), and found immediate relief."

*Dr. Farley:* "*Anac.* has desire to swear with his ailment."

The next business was the reading of paragraph 245 of the *Organon* by Dr. George H. Clark, who presented a brief paper on "The Repetition of the Dose." The Doctor said Hahnemann's views were not to be improved upon, so that there was no necessity for discussing the question. Failures were due to neglect to adhere strictly to his advice.

Discussion:

*Dr. Clark:* "Administer *first* the medicine that is strongly homœopathic, then wait, even though it does not show an immediate improvement."

*Dr. Edmund J. Lee:* "The question whether to repeat the old medicine or give another amounts in either case to a new prescription."

Dr. Clark was granted time to read an extract from Gregg's "Diphtheria" relative to the point under debate.

Discussion:

*Dr. Lee:* "Dr. Pomeroy once told me that he had made his best cures through mail, as he would then send the needed prescription and be compelled to await report without chance to change the remedy. I know of a lady who had her eyes examined by a celebrated oculist for cataract, and who, on being told by him that an operation was necessary, consulted Dr. Lippe, who cured her with one dose of *Calcarea.* The allopath, on hearing that she had been cured, said that he had made a false diagnosis."

*Dr. Clark:* "Dr. Fellger cured a case of congenital cataract with *Nat. mur.*"

*Dr. Lee:* "A child had convulsions and was better by being carried about quickly in each attack. One dose of *Cham.* caused it to urinate freely twice (which it had not done at all for some time), and I think, from other indications, would have cured the case had not the family been persuaded to change to allopathy, and the child died."

*Dr. Guernsey:* "*Arsen.* has relief of symptoms from being carried rapidly."

*Dr. C. Carleton Smith:* "A difficult thing to contend with is the repetition of the dose. I have no fear to leave a patient on the single dose any time. If the physician has any such doubt whether to give medicine continuously or wait, he should always do the latter. Since doing this myself I have lost fewer cases. If the patient seems to need a repetition, have a powder dissolved in water and give a few doses in quick succession and then wait on Sac. lac."

*Dr. Clark:* "That amounts to a single dose."

*Dr. Smith:* "I saw a case once where *Bella.* was clearly indicated, and learning that the previous doctor had used that remedy continuously, gave a few doses of the CM potency. After taking the third dose it showed marked improvement and needed nothing more. The cases I do not have access to I cure the best. I once cured a case of long standing catarrh with one dose of *Kali bichrom.*$^{mm}$. The patient was a clergyman who had written me that he had been compelled to leave town every year on account of it. Had I seen him I might have prescribed a medicine to be taken repeatedly, but as it was I sent the one dose, and not hearing from him before it had had full chance to act it worked a perfect cure. A case of gonorrhœa, which had been under allopathic treatment for some time, came to me and I gave one dose of *Puls.*$^{7mm}$, which caused a resumption of the discharge and subsequent cure in ten days. In regard to Hahnemann's idea of olfaction, I would say that I saw Hering cure a case of convulsions with a single inhalation of *Opium.*"

*Dr. John V. Allen*, as per appointment, read an excellent paper on typhoid fever, having, as an appendix, a repertory.

Discussion:

*Dr. Allen:* "I have used milk diet mainly."

*Dr. Guernsey:* "I frequently use unfermented wine. There are three preparations on the market—Dr. Welch's, of Vineland; Spears', of Passaic, and Dr. Tuller's, of Vineland. The latter is not sweet enough to suit most patients."

*Dr. Clark:* "If it contains sugar it will cause fermentation in the stomach and trouble will follow."

*Dr. Guernsey:* "I have not had the least trouble in that way and use it a great deal."

The next business was election of new members—nine associates being elected and two names added to the honorary list.

The following appointments were made for the next meeting: *Materia Medica*, Dr. George H. Clark; *Organon*, Dr. R. B. Johnstone; *Original Paper*, Dr. Robert Farley.

THE HOMŒOPATHIC PHYSICIAN was made the organ of the Association, and all papers ordered to be sent to its editors for their acceptance or rejection at option.

Dr. Guernsey presented a number of papers published by the "London Anti-Vaccination League" (which had been furnished him by Dr. Stow, of Mexico, N. Y.), and offered a resolution condemning vaccination, which was tabled for further discussion. WM. JEFFERSON GUERNSEY, M. D., *Secretary*.

